# A bayesian shared component spatial modeling approach for identifying the geographic pattern of local associations: a case study of young offenders and violent crimes in Greater Toronto Area

**DOI:** 10.1186/s40163-024-00235-5

**Published:** 2024-10-30

**Authors:** Jane Law, Abu Yousuf Md Abdullah

**Affiliations:** 1 School of Planning, University of Waterloo, 200 University Avenue West, Waterloo, ON N2L 3G1 Canada; 2 School of Public Health Sciences, University of Waterloo, Waterloo, ON Canada

**Keywords:** Bayesian, Shared component spatial modeling, Joint models, Shared risk, Young offenders, Violent crimes, Spatial association, Association mapping

## Abstract

**Background setting:**

Traditional spatial or non-spatial regression techniques require individual variables to be defined as dependent and independent variables, often assuming a unidirectional and (global) linear relationship between the variables under study. This research studies the Bayesian shared component spatial (BSCS) modeling as an alternative approach to identifying local associations between two or more variables and their spatial patterns.

**Methods:**

The variables to be studied, young offenders (YO) and violent crimes (VC), are treated as (multiple) outcomes in the BSCS model. Separate non-BSCS models that treat YO as the outcome variable and VC as the independent variable have also been developed. Results are compared in terms of model fit, risk estimates, and identification of hotspot areas.

**Results:**

Compared to the traditional non-BSCS models, the BSCS models fitted the data better and identified a strong spatial association between YO and VC. Using the BSCS technique allowed both the YO and VC to be modeled as outcome variables, assuming common data-generating processes that are influenced by a set of socioeconomic covariates. The BSCS technique offered smooth and easy mapping of the identified association, with the maps displaying the common (shared) and separate (individual) hotspots of YO and VC.

**Conclusions:**

The proposed method can transform existing association analyses from methods requiring inputs as dependent and independent variables to outcome variables only and shift the reliance on regression coefficients to probability risk maps for characterizing (local) associations between the outcomes.

**Supplementary Information:**

The online version contains supplementary material available at 10.1186/s40163-024-00235-5.

## Introduction

Most crime problems warrant a thorough understanding of the relationship between the factors causing or influencing the crime patterns. Unfortunately, these relationships or associations between real-life factors are often highly complex and may not readily adhere to the linear and parametric assumptions of traditional modeling techniques (Law & Abdullah, 2022). Furthermore, real-life events, such as crime, often show distinct spatial patterns, which can be further influenced by various extraneous spatial and non-spatial factors (Anselin, 1995, 2019; Miller, 2004). Therefore, modeling accuracies can be severely compromised without applying prior knowledge of the dependent and independent nature of the variables and without adequate adjustments of the other exogenous variables.

However, prior knowledge about some complex crime phenomena can be difficult to obtain. For example, it is often impossible to understand whether the high presence of young offenders (YO) is contributing to the rise of violent crimes (VC) in an area or whether it is the high occurrence of VC in an area that is encouraging the rise of YO. Past research found that offenders who engaged in crimes during their youth were at least twice at the risk of committing VC during adulthood than offenders who did not engage in any crime during their youth (Loeber & Farrington, 2000). There is further evidence that YO may become chronic adult offenders of VC in the future (Law & Quick, 2013). While these findings suggest that YO could be an important predictor of VC, other research reported that the spatial variation in risk for YO could be explained more (YO: 48% vs. VC:24%) by the cooccurrence of YO and VC (Law & Abdullah, 2022). This suggests that VC can also act as a critical factor in determining the spatial distribution of YO in an area, showing that the relationship between YO and VC could be bidirectional in real life, where the presence of YO can potentially lead to the occurrence of VC and, alternatively, the VC occurring in an area can give rise to youths becoming YO.

Conventionally, spatial regression analyses have been used to understand multiple outcomes such as VC and YO (Cahill & Mulligan, 2007; Messner et al., 2013; Wang et al., 2019). However, several limitations arise when applying these techniques to study crime, such as the association between YO and VC. First, the traditional spatial regression models, such as the spatial lag or error models, provide limited information on the spatial interaction between YO and VC, which can be useful for crime management practices. For an analysis studying the association between YO (dependent) and VC (independent), the spatial lag term will only describe to what extent the average prevalence of YO in the neighbors can explain the actual prevalence of the offenders in a particular location (Anselin & Bera, 1998; Anselin et al., 2000). In contrast, the spatial error model will perceive spatial interactions between YO and VC as a nuisance and adjust its effect on the model through the error term (Ward & Gleditsch, 2018). Thus, neither the lag nor the error term will have any intuitive meaning that could be used for crime management applications as they just indicate that the observations are correlated due to the influence of unmeasured factors, which, for reasons or factors unknown, are also correlated based on distances among the observations (Anselin et al., 2000; Tita & Radil, 2010; Ward & Gleditsch, 2018).

Second, considering local regression methods, such as GWR, separate regression models can be fitted for each YO observation using VC as the predictor. As a result, although local patterns could be better identified, the global relationship between the dependent and independent variables can be difficult to ascertain. In other words, how the YO and VC are interlinked throughout the study area may not be sufficiently understood. Furthermore, these local models may produce edge effects and biased parameter estimates when the data is sparse, or the independent variable (here, for example, the VC) shows large geographical variations (Leong & Yue, 2017). Even in the more advanced versions of GWR, such as the geographically weighted Poisson regression (GWPR) or the geographically weighted logistic regression (GWLR), the overdispersion and zero count problems in the observational data may become extremely difficult to address (Chen et al., 2020). Third, and most importantly, these modeling techniques (spatial lag, spatial error, or GWR) will require variables to be clearly defined as either dependent or independent variables, which, as mentioned earlier, may not always be easy to discern since YO and VC can both influence each other’s spatial distribution.

Due to these limitations of the conventional methods, several advanced spatial regression techniques have been proposed in crime studies. The spatial frequentist techniques include the zero-inflated negative binomial model (Liu et al., 2018; Swartout et al., 2015), geographically weighted negative binomial regression (GWNBR) (Chen et al., 2020; Wang et al., 2017), spatial Durbin (R. P. Haining & Li, 2020), and spatial spline regression models (Sangalli et al., 2013). Additionally, with the advancement of computational power, Bayesian spatial techniques have gained considerable popularity, such as the Bayesian Poisson hierarchical regression (Law & Haining, 2004; Law & Quick, 2013; Persad, 2020; Quick et al., 2017), Bayesian semiparametric joint quantile regression (Bresson et al., 2021; Chen & Tokdar, 2021; Jang & Wang, 2015; Kottas & Krnjajić, 2009), Bayesian cross-classified multilevel spatial (and temporal) modeling (Quick, 2019) and Bayesian spatial network learning (Baumgartner et al., 2005; Mahmud et al., 2016). Each of these techniques is applied considering different aspects of crime and poses its own advantages and disadvantages.

Considering the research gap and the complexities associated with multivariate modeling of crime variables, this study, through applying crime data from the York Region in Canada, aims to propose the application of the Bayesian Shared Component Spatial (BSCS) modeling technique to analyze the spatial dynamics of crime, specifically the association between YO and VC. Our choice of BSCS modeling is governed by several critical modeling advantages. First, Bayesian spatial techniques help integrate our prior beliefs on VC and YO with the observed data and treat all unknown parameters (such as regression coefficients, $\:{\beta\:}_{k}$) to be associated with a probability distribution (Haining et al., 2009; Haining & Li, 2020; Law & Haining, 2004). The Bayesian approach thus uses multivariate prior probability distributions to impose a spatial dependence structure on the parameters to be estimated and allow adjustments owing to spatial variations (autocorrelation and overdispersion) in the observed data (R. P. Haining & Li, 2020). This, in turn, helps improve the precision of the parameter estimates and facilitates borrowing information from observations in adjacent areas and correlated outcomes, which can be extremely useful in areas with sparse data.

Moreover, crime is a multidimensional phenomenon where many socioeconomic and sociocultural factors are at interplay (Berk & MacDonald, 2009; Brantingham et al., 2017; Bursik Jr, 1988; Chopin & Caneppele, 2019; Wortley & Townsley, 2016). Complex spatial and non-spatial interactions and correlations between the confounding variables must be addressed when developing a model to assess the spatial relationship between YO and VC. In this regard, BSCS modeling, as a joint-modeling technique, allows adjusting for the multidimensionality associated with the main and higher-order interaction effects of the studied outcomes (YO and VC) and any confounders (Papageorgiou et al., 2015). Lastly, the use of BSCS modeling allows the realization of three major spatial processes within the model architecture (Cesaroni & Doob, 2020): first, the youth crime, which can be modeled as a function of the spatial processes occurring across different neighborhoods in the study area; second, the influence from the putative risk factors that affect the distribution of YO and VC (Law & Quick, 2013; Law et al., 2015, 2020), and lastly, the influence of non-spatial protective measures, such as the youth justice system that responds to the spatially varying occurrence of violent youth crimes (Cesaroni & Doob, 2020). Hence, the outputs of BSCS models have an intuitive meaning that can be used for assessing crime risks, mapping shared and YO- or VC-specific hotspots, and understanding high-priority areas for crime management interventions that can simultaneously target to reduce risk from YO and VC.

The problem studied in our research engages with two crime management policy questions in Canada, “Where and how are violent crimes related to young people?“. In 2019, the police-reported Violent Crime Severity Index (VCSI) in Canada rose by 7% from the preceding year, with 77,200 youth accused of a criminal offense (Moreau, 2019). Unfortunately, due to the limitations of statistical techniques to model the complex real-life interactions between VC, YO, and the associated risk factors (Berk & MacDonald, 2009; Brantingham et al., 2017; Wortley & Townsley, 2016), a paucity of knowledge exists on how YO and VC co-exist in Canada. This study is a continuation and extension of the work by Law and Quick (2013) and specifically aims to demonstrate the application of BSCS modeling for identifying the geographic and null (zero) association between at least two variables of interest (will be termed as ‘outcomes’ for BSCS models), which are spatially interlinked and are subjected to the effects of exogenous variables. The previous study by Law and Quick (2013) explored the links between YO and social disorganization, whereas this study will focus on analyzing the relationship between YO and VC while adjusting the social disorganization factors identified in the earlier study as putative risk factors in the models. In addition, the potential and benefits of the proposed approach of employing BSCS modeling to simultaneously analyze the association between multiple outcomes and mapping high-risk zones are explored and compared against the common spatial regression techniques. Specifically, this research aims to answer two research questions:

- Do areas with more violent crime also have more young offenders?
- Can the Bayesian shared component spatial modeling technique be used to understand how the YO and VC are spatially associated after adjusting for the influence of potential confounders?

In the next sections, we introduce the modeling strategies involved in applying BSCS models to identify the association between multiple outcomes and discuss the theoretical and statistical basis of the work. Afterward, we present the results of comparing BSCS with non-BSCS methods, and finally, we critically discuss the findings with regard to applications in crime management practices and report the strengths and limitations of this work.

## Methods

### Study area and data

The study area for this research is the York Region of Southern Ontario in Canada, with a population of 892,712 in 2006. The geographical unit of study is the census tract or the Dissemination Area (DA) level, comprising 1128 DAs. The DAs are defined as having a uniform standard population ranging from 400 to 800 persons and covering the entire region of Canada (Statistics Canada, 2018). The data for young offenders and violent crimes was obtained from the York Regional Police Department (YRPD) from January 1, 2006, to December 31, 2007. The term ‘young offenders’ refers to male and female offenders aged under 20 years and involved in one or more crimes during the study period. The ‘violent crimes’ refer to crimes defined using the Uniform Crime Reporting (UCR) codes (1000 $\:\le\:\:$UCR codes $\:<\:$2000) and include crimes causing deaths (such as first-degree and second-degree murders), assaults (such as bodily harm, assaults using a weapon and aggravated assaults) and sexual assaults (such as threatened sexual assaults using a weapon and aggravated sexual assaults) (Uniform Crime Reporting (UCR), 2006).

The YO engaged in more than one charge was counted only once. Contrastingly, the number of VC towards each victim in each DA was counted separately. Hence, if one young offender had committed two incidents of VC for two different victims, this was counted as two separate cases of VC. However, for robbery, one incident by multiple offenders was counted as a single incident of VC because robbery generally involves multiple offenders and, therefore, may lead to an overstatement of its occurrence when counted multiple times for multiple offenders or victims (Law et al., 2015). The street addresses for each offender record were available with geographic coordinates (x, y), which were geocoded using ArcGIS 10.0 with a success rate of 98%. A point-in-polygon method was applied to calculate the total counts of YO and violent offenses in each DA during the 2006–2007 period. The population at risk was estimated as the total number of residents aged below 20 years per DA, and the indirect standardization method was applied to estimate the expected counts of YO and VC in each area. The average trend of VC (2006 to 2007) in the entire York Region was multiplied by the residential population (based on the Census 2006) in each DA to estimate the expected count of VC. In contrast, the expected number of YO was estimated by standardizing age and sex groups under 20 years of age.

The data for demographic and socioeconomic variables were retrieved from Statistics Canada and were used as potential confounders in the regression models. Based on the literature review, fifteen variables that could help explain the location of YO in the study area were considered in the original study by Law and Quick (2013). The study applied various non-spatial and spatial regression techniques to analyze the links between YO and the fifteen variables. The findings suggested that the three most significant explanatory variables that can help explain the distribution of YO in the DAs are the index of ethnic heterogeneity, residential mobility (1-year moving rate), and percentage of residents receiving government transfer payments (Law & Quick, 2013). Consequently, these three significant variables have been considered for this study. For more details on all fifteen variables and the methods employed to select the three variables, please consult Law and Quick (2013).

### Modeling strategies: Non-BSCS vs. BSCS models

**Fig. 1:**
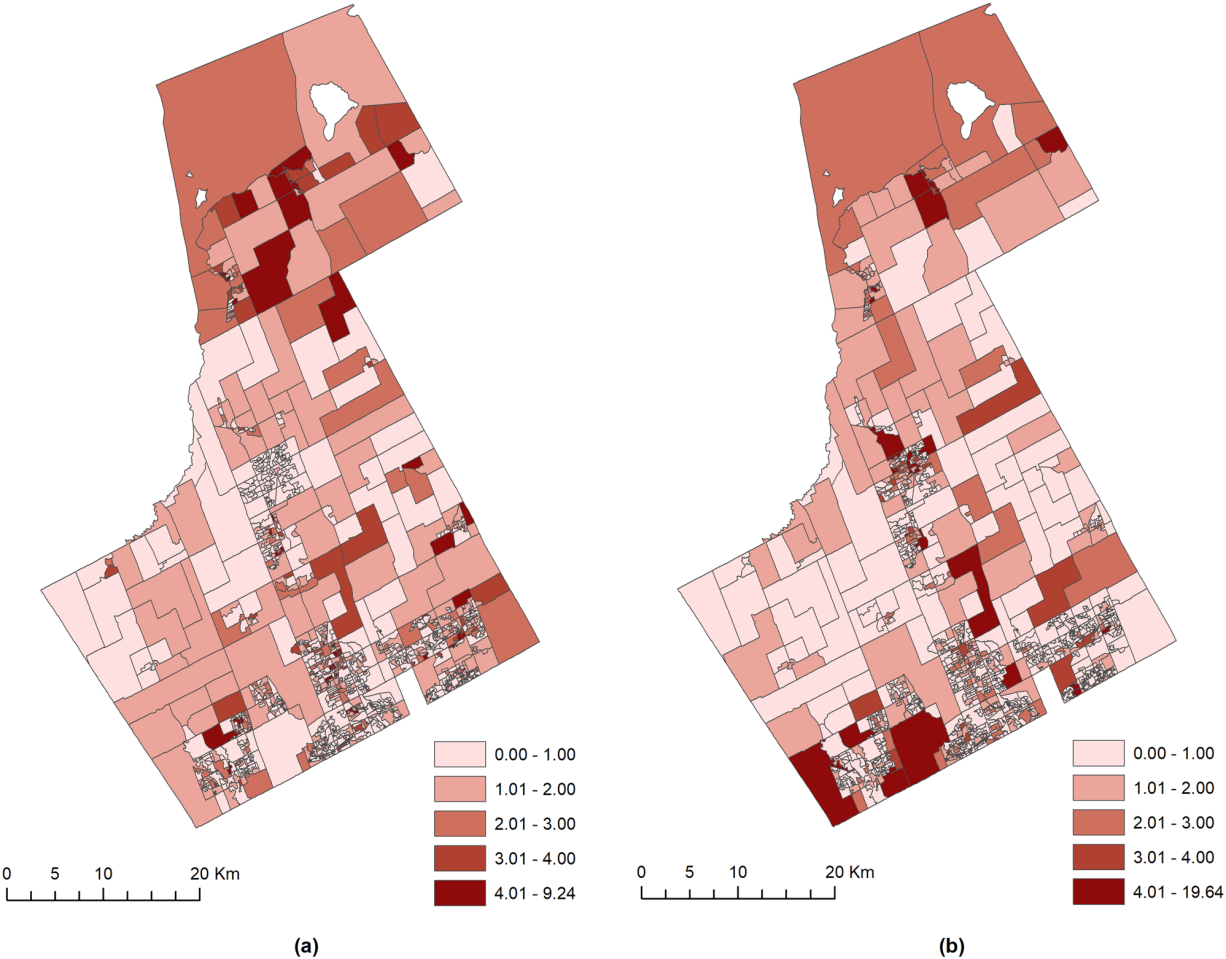
The standardized risk ratio (crude relative risk) of (**a**) YO and (**b**) VC

The crude relationship between the YO and VC, based on which the modeling strategies were developed, was explored by mapping the geographic distribution of the standardized ratio of the risk of YO and VC. The standardized ratio of risk (crude relative risk) was estimated using the ratio between observed and expected counts of YO and VC (Law & Quick, 2013; Law et al., 2020). Figure 1 shows the crude relative risk of YO and VC, which suggests a potential association between YO and VC. This is because most (but not all) DAs with higher risk from YO (Fig. 1a) also exhibited higher risk from VC (Fig. 1b).

Therefore, our modeling aimed to identify three distinct types of risk areas: areas where (i) both YO and VC risks are high or the common/shared risk areas, (ii) only YO risks are high, and (iii) only VC risks are high. Thus, the modeling strategy (explained in detail in Additional File 1) was based on the assumption that the (i) common/shared high-risk areas may indicate a (positive) association between YO and VC, and areas of (ii) only high risk of YO and (iii) only high risk of VC may indicate no (positive) association. For this purpose, three models were developed:

#### Model 1

A Bayesian Poisson log-linear model with YO as the outcome variable and the three covariates (the index of ethnic heterogeneity, residential mobility, and percentage of residents receiving government transfer payments). The Eq. 1 defined Model 1.

1 $$\:{\text{l}\text{o}\text{g}\:[\lambda\:}_{i}]=\:{\text{l}\text{o}\text{g}\:[E}_{i}]\:+\:{\beta\:}_{0}+\:{\beta\:}_{1}{X}_{1i}+\:{\beta\:}_{2}{X}_{2i}+\:{\beta\:}_{3}{X}_{3i\:}+\:{s}_{i}\:+\:{u}_{i}$$

#### Model 2

A Bayesian Poisson log-linear model with YO as the outcome variable and the above three covariates (Model 1) and VC as the fourth covariate, as explained by Eq. 2.

2 $$\begin{aligned}{\text{l}\text{o}\text{g}\:[\lambda\:}_{i}]&=\:{\text{l}\text{o}\text{g}\:[E}_{i}]\:+\:{\beta\:}_{0}+\:{\beta\:}_{1}{X}_{1i}\cr&\quad +\:{\beta\:}_{2}{X}_{2i}+\:{\beta\:}_{3}{X}_{3i\:}\cr&\quad +\:{\beta\:}_{4}{X}_{4i\:}+\:{s}_{i}\:+\:{u}_{i}\end{aligned}$$

Here, for Models 1 and 2, the observed counts of the YO, $\:{O}_{i}$, in each DA, *i* (where, *i* = 1, 2,…1128), were modeled using the Poisson distribution, $\:{O}_{i}\sim\:Poisson\left({\lambda\:}_{i}\right).$ The parameter $\:{\lambda\:}_{i}$ of the distribution represented the expected value of $\:{O}_{i}$. $\:{E}_{i}$ represented the area-specific expected count of YO. The $\:{X}_{1i}$, $\:{X}_{2i}$, $\:{X}_{3i}$ and $\:{X}_{4i}$ represent the index of ethnic heterogeneity, residential mobility, percentage of residents receiving government transfer payments, and the rate of VC in each DA, respectively. The $\:{\beta\:}_{k}$, where $\:k\:=\:1,\:2,\:3,\:\text{a}\text{n}\text{d}\:4$, represent the regression coefficients. The random effect terms, $\:{s}_{i}\:\text{a}\text{n}\text{d}\:{u}_{i}$, captured the spatially structured and unstructured risks, respectively, due to the unmeasured or latent covariates. For more details, please refer to the Additional File 1.

#### Model 3

A Bayesian shared component model with YO and VC as outcome variables and no covariates (Eqs. 3 and 4).

3 $${\text{log}({{\uplambda\:}}_{i1}}_{\:})=\text{log}{(E}_{i1})\:+\:{\alpha\:}_{1}+\:\delta\:{\theta\:}_{i}+{s}_{i1}+\:{u}_{i1}$$

4 $$\:{\text{log}({{\uplambda\:}}_{i2}}_{\:})=\text{log}{(E}_{i2})\:+\:{\alpha\:}_{2}+\frac{1}{\delta\:}{\theta\:}_{i}+{s}_{i2}+\:{u}_{i2}$$

#### Model 4

A Bayesian shared component model with YO and VC as outcome variables and the above three covariates in Model 1 (Eqs. 5 and 6).

5 $$\begin{aligned}{\log({{\lambda\:}}_{i1}}_{\:})&=\log{(E}_{i1})\:+\:{\alpha\:}_{1}+\:\delta\:{\theta\:}_{i}+\:{\beta\:}_{0}+\:{\beta\:}_{1}{X}_{1i}\cr&\quad +\:{\beta\:}_{2}{X}_{2i}+\:{\beta\:}_{3}{X}_{3i\:}+{s}_{i1}+\:{u}_{i1}\end{aligned}$$

6 $$\begin{aligned}{\log({{\lambda\:}}_{i2}}_{\:})&=\log{(E}_{i2})\:+\:{\alpha\:}_{2}+\frac{1}{\delta\:}{\theta\:}_{i}+\:{\beta\:}_{0}\cr&\quad +\:{\beta\:}_{1}{X}_{1i}+\:{\beta\:}_{2}{X}_{2i}+\:{\beta\:}_{3}{X}_{3i\:}+{s}_{i2}+\:{u}_{i2}\end{aligned}$$

Here, the observed counts, $\:{O}_{ik}$, where $\:k=\:1\:\text{a}\text{n}\text{d}\:2\:$ for the YO and VC, respectively, were modeled using the Poisson distribution as $\:{O}_{ik}\sim\:Poisson\left({\lambda\:}_{ik}\right)$. The parameter $\:{\lambda\:}_{ik}$ of the distribution represented the expected value of $\:{O}_{ik}$. The $\:{\alpha\:}_{1}$ and $\:{\alpha\:}_{2}$ are the specific intercepts for YO and VC, respectively, and give the baseline or average risks from YO or VC in an area. The common spatial risk shared by YO and VC in an area *i* was given by $\:{\theta\:}_{i}$. The non-shared or the type-specific risks for YO and VC were modeled using one spatially structured random effect term, $\:{s}_{ik}\:,\:$and one non-spatial random effect term, $\:{u}_{ik}$. The unique contribution from YO and VC in the shared risk and the risk gradient of the shared component was modeled as a scaling parameter $\:\delta\:$ (where $\:\delta\:$ > 0). A value of $\:\delta\:$ near to one would indicate that the YO and VC have similar contributions to the shared pattern, whereas a large positive value of $\:\delta\:$ would indicate that the YO contributed more to the shared risk than the VC and has a stronger spatial association with the shared component compared to VC. For more details, please refer to Additional File 1.

In our modeling strategy, Model 1 was set as the control against which the findings from Model 2 could be compared to elucidate how important VC is for explaining the distribution of YO in the DAs. Model 2 directly addressed our first research question: do DAs with more VC also have more YO? Models 3 and 4 attempted to answer the second research question: Can the BSCS modeling technique be used to understand how the YO and VC are spatially associated?

Moreover, Models 3 and 4 aim to demonstrate how the spatial association between YO and VC could be studied without differentiating YO and VC into dependent and independent variables. Comparing the findings from Model 4 with Model 2 can show how the results of association analyses may differ when non-BSCS and BSCS modeling approaches are used with the same set of covariates.

A detailed description of the model construction methodologies, implementation techniques, and assessment criteria can be found in Additional File 1.

### Relative risk assessment and hotspot mapping using BSCS models

The shared risks of YO and VC were assessed using the posterior mean values of the $\:{\text{e}\text{x}\text{p}(\theta\:}_{i})$ from the Bayesian models. Whereas the remaining (unexplained) and type-specific spatial and non-spatial risks of YO were estimated using $\:{\text{e}\text{x}\text{p}(s}_{i1})$ and $\:{\text{e}\text{x}\text{p}(u}_{i1})$, respectively. The statistical significance of the risk values was assessed at the 95% credible interval (CI), with risk values greater than 1 at the 95% CI considered as high risk from the YO.

The posterior probability of $\:{\text{e}\text{x}\text{p}(\theta\:}_{i})$ being greater than one (Pr(exp($\:{\theta\:}_{i}$) > 1 | data)) was recorded to map the YO-VC shared hotspot areas. Similarly, the posterior probability of the $\:{\text{e}\text{x}\text{p}(s}_{i1}+{u}_{i1})$ greater than 1 (Pr(exp($\:{s}_{i1}\:+\:{u}_{i1}$) > 1 | data)) was used to identify the YO-specific hotspots.

Finally, we recorded the fraction of total variation in relative risk of YO that was attributable to the shared component or the $\:\text{V}\text{a}\text{r}\:\left(\delta\:{\theta\:}_{i}\right)/\:\left(\text{V}\text{a}\text{r}\:\right(\delta\:{\theta\:}_{i})+\text{V}\text{a}\text{r}\left({s}_{i1}\right)+\text{V}\text{a}\text{r}\left({u}_{i1}\right))$. This ratio of the empirical variance of $\:\delta\:{\theta\:}_{i}$ and the sum of the empirical variances of$\:\:\delta\:{\theta\:}_{i},{s}_{i1}\:\text{a}\text{n}\text{d}\:{u}_{i1}$ was used to understand the importance of the spatial shared component in identifying high-risk zones that could be targeted to co-manage the risks from YO and VC.

## Results

### Modeling the association between YO and VC: the non-BSCS and BSCS models

The results of the Bayesian Poisson log-linear spatial regression (Models 1 and 2) and the Bayesian shared component spatial modeling analyses (Models 3 and 4) are tabulated in Table 1. The results indicate that the index of ethnic heterogeneity ($\:{\beta\:}_{1}$), residential mobility ($\:{\beta\:}_{2}$), and the percentage of residents receiving government transfer payments ($\:{\beta\:}_{3}$) are all significantly associated with the YO variable in Models 1 and 2. More importantly, in Model 2, the VC is found to be significantly and positively associated with the YO after adjusting for the influence of ethnic heterogeneity, residential mobility, and government transfer payments. This indicates that the VC in an area can significantly influence the distribution of YO, and the DAs with more VC had indeed contained more YO. Model 2 had a slightly lower DIC value (DIC = 3359.37) compared to Model 1 (DIC = 3361.16), indicating that the two models were very similar in terms of model fit.

**Table 1:** The results from the Bayesian Poisson log-linear and Bayesian shared component spatial models

	Model 1	Model 2	Model 3	Model 4
β_1_: ethnic heterogeneity	1.406 (0.823, 2.006)	1.322 (0.718, 1.945)	NA	1.250 (0.642, 1.864)
β_2_: residential mobility	0.008 (0.001, 0.015)	0.008 (0.001, 0.015)	NA	0.0071 (-0.0003, 0.0147)
β_3_: government transfer payments	0.031. (0.015, 0.047)	0.028 (0.012, 0.045)	NA	0.024 (0.007, 0.041)
β_4_: violent crime	NA	0.041 (0.002,0.078)	NA	NA
δ scaling parameter	NA	NA	1.175 (0.799, 1.645)	1.130 (0.634, 1.857)
Fraction of total variation in relative risk of YO that was explained by the shared component	NA	NA	0.492 (0.151, 0.913)	0.361 (0.047, 0.885)
DIC (YO)	3361.16	3359.37	3341.53	3323.65

The BSCS models, Models 3 and 4, jointly analyzed the YO and VC as multiple outcomes. The values for the scaling parameter of the risk, $\delta$, were 1.175 (95% CI: 0.799, 1.645) and 1.130 (95% CI: 0.634, 1.857) for Models 3 and 4, respectively. Additionally, and in contrast to Models 1 and 2, only the ethnic heterogeneity and government transfer payment covariates were found to be statistically significant in Model 4. As Model 4 comprises the three covariates, its shared component value is adjusted for the effects of potential confounders that could influence the distribution of YO and VC in the study area. Comparing the DICs of YO, which is an outcome variable for all four models, indicates that Model 4 fits the YO data best. Also, a difference of DICs of 35.72 (3359.37–3323.65) between Models 2 and 4 clearly indicates that the BSCS fits the YO data better than the non-BSCS model. Models 2 and 4 analyzed the association between the YO and VC after adjusting for the putative measured and unmeasured risk factors in the study area.

### Relative risk assessment and hotspot mapping

The relative risk assessment was carried out to understand the shared risk of YO and VC. The fraction of total variation in the relative risk of YO that was attributable to the shared component gave the strength of the spatial association between YO and VC. Table 1 suggests that when no covariates are adjusted in the model (Model 3), about 49.2% of the total variation in the relative risk from YO in the study area could be significantly explained by the YO-VC shared risk. In contrast, about 36.1% of the total variation in relative risk from YO could be explained by the shared risk when the covariates are included in the model (Model 4).

**Fig. 2:**
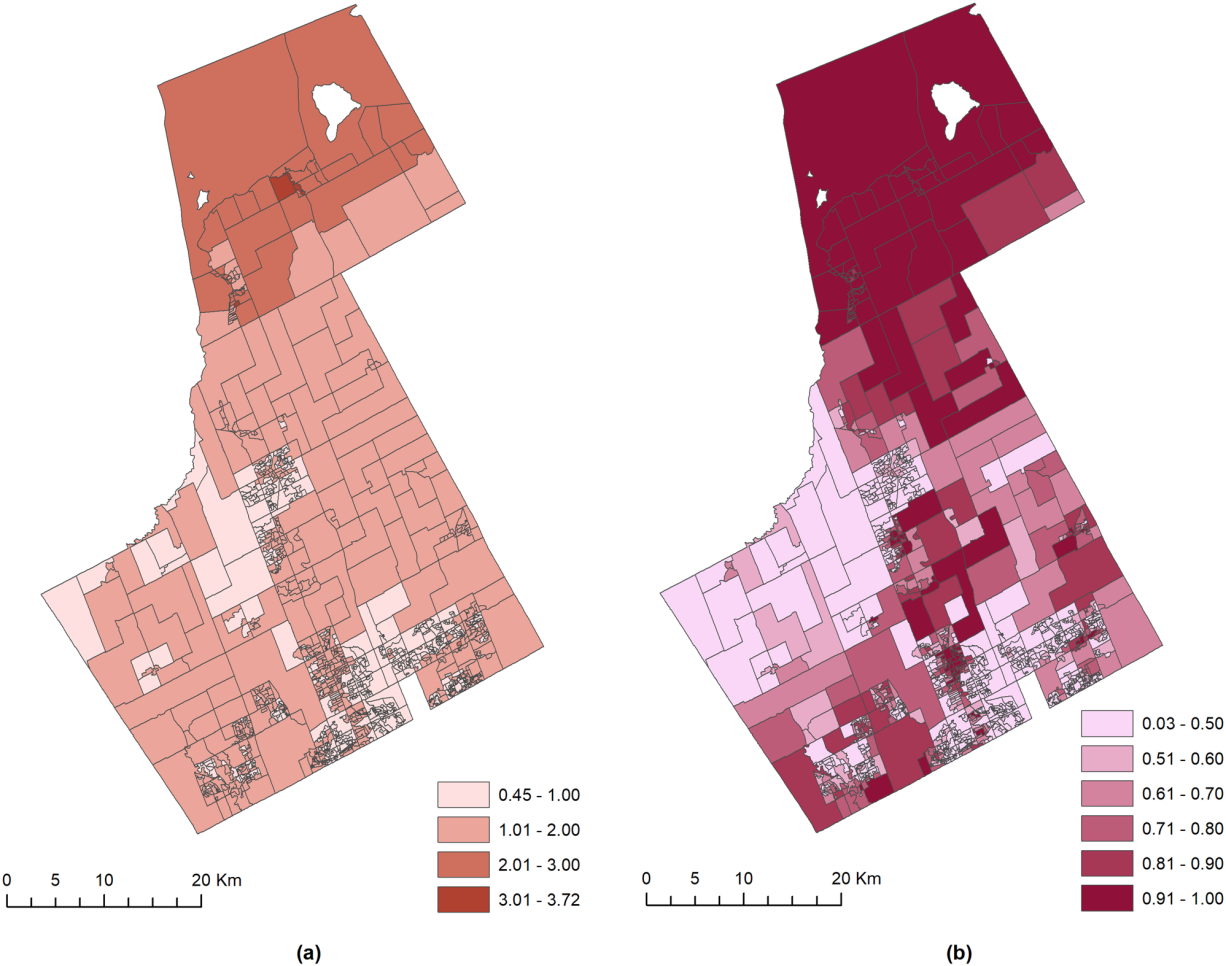
The spatial distribution of (**a**) the shared risk of YO-VC ($\:{\text{e}\text{x}\text{p}(\theta\:}_{i})$) and (**b**) the YO-VC shared hotspots (Pr(exp($\:{\theta\:}_{i}$) > 1 | data))

**Fig. 3:**
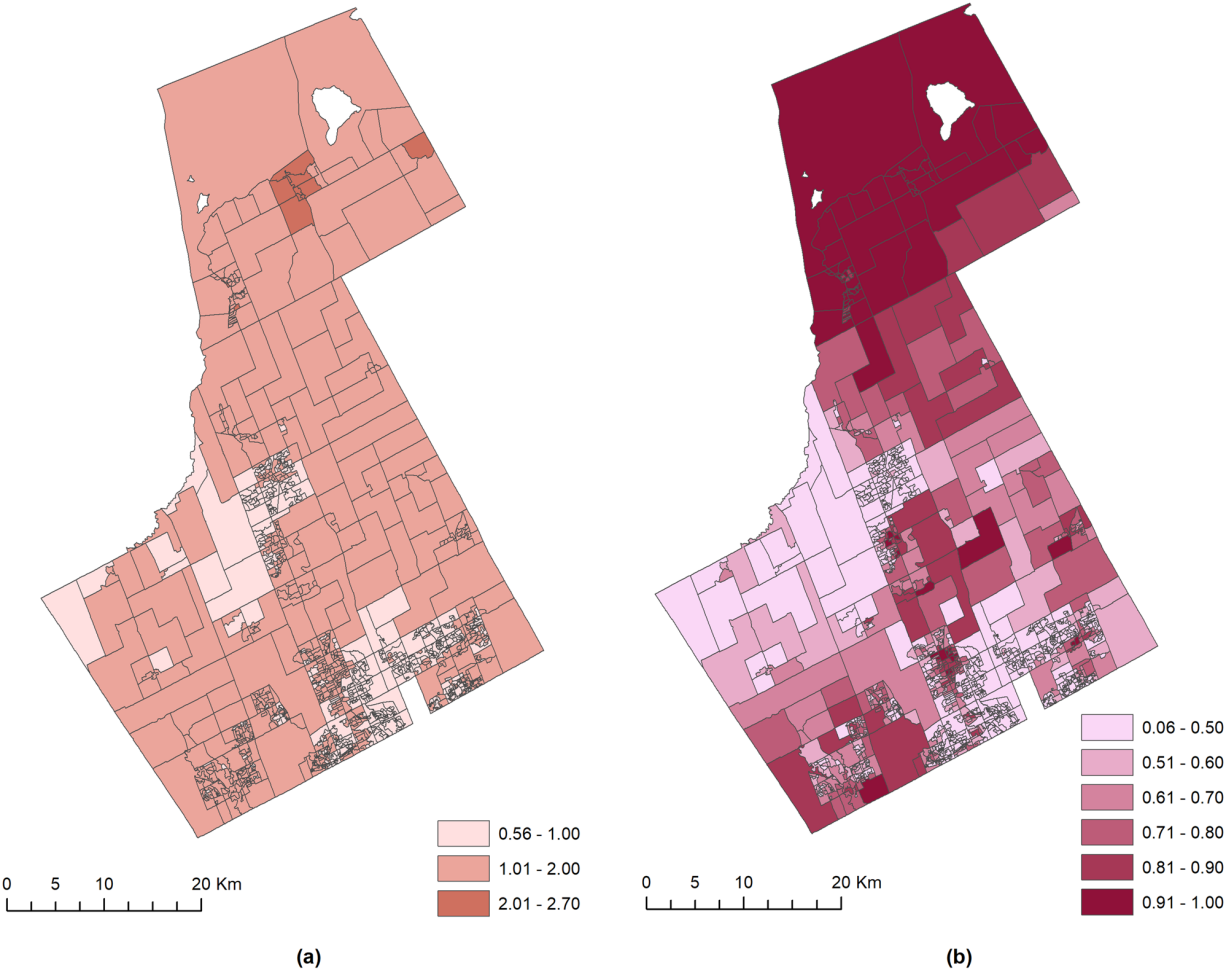
The spatial distribution of (**a**) the shared risk of YO-VC ($\:{\text{e}\text{x}\text{p}(\theta\:}_{i})$) and (**b**) the YO-VC shared hotspots (Pr(exp($\:{\theta\:}_{i}$) > 1 | data)), after adjusting for ethnic heterogeneity, residential mobility, and government transfer payments

The shared risk before and after adjusting the potential confounders or putative risk factors in the BSCS models are presented in Figs. 2 and 3, respectively. The shared component risk ($\:{\text{e}\text{x}\text{p}(\theta\:}_{i})$) and probability (Pr(exp($\:{\theta\:}_{i}$) > 1 | data)) maps from Model 3 in Fig. 2 show that the northern portion of the York region was the most vulnerable to the risk of YO and VC (Fig. 2a) and was a crime hotspot (Fig. 2b). The northern DAs comprised a shared risk that ranged between 2.00 and 3.72, with most DAs comprising a risk probability of 0.91 and above. Figure 2a further illustrates that a majority of the DAs in the central region comprised moderately low risk $\:{(1<\text{e}\text{x}\text{p}(\theta\:}_{i})\le\:2)$ from both YO and VC, with few DAs in the central-western part having a very low risk $\:{(1>\text{e}\text{x}\text{p}(\theta\:}_{i}\left)\right)$. Interestingly, Fig. 2b refined the risk map further and showed some divergent findings. For example, in the south-central and south-western regions where the DAs had a moderately low risk, the probability of risk of crime was extremely high (0.9 > Pr(exp($\:{\theta\:}_{i}$) ≥ 1). Generally, these hotspots having a high probability, such as > 0.9, can be interpreted as areas of high risk from YO and VC and where the associations between the two outcomes are the strongest. Similarly, areas with a probability < 0.8 in the hotspot map can be interpreted as areas with relatively weaker associations, with a minimal association in areas having probability values < 0.5.

The shared component risk ($\:{\text{e}\text{x}\text{p}(\theta\:}_{i})$) and probability (Pr(exp($\:{\theta\:}_{i}$) > 1 | data)) maps from Model 4 in Fig. 3 illustrate the YO-VC shared risks and hotspots after adjusting for the effects of ethnic heterogeneity, residential mobility, and government transfer payments. Figure 3a shows that the inclusion of the three covariates substantially stabilized the risk values and probability estimates, as observed from the reduced variations in the range of risk and probability values. Figure 3a shows that the northern region was at moderately low risk, with values ranging from 1.01 to 2, when compared with Fig. 2a, having risk values above 2. However, both the shared risk maps in Figs. 2a and 3a show that the majority of the DAs had moderately low risk. Therefore, the inclusion of the covariates appears to have the most discernible impact on the extreme values of the shared risk. Alternatively, the hotspot mapping in Fig. 3b did not exhibit any distinctive difference when compared with Fig. 2b. Although the probability of YO-VC shared risk for some DAs in the central and central-north areas was lower in Fig. 3b, a majority of the probability values remained unchanged after the inclusion of the three covariates.

**Fig. 4:**
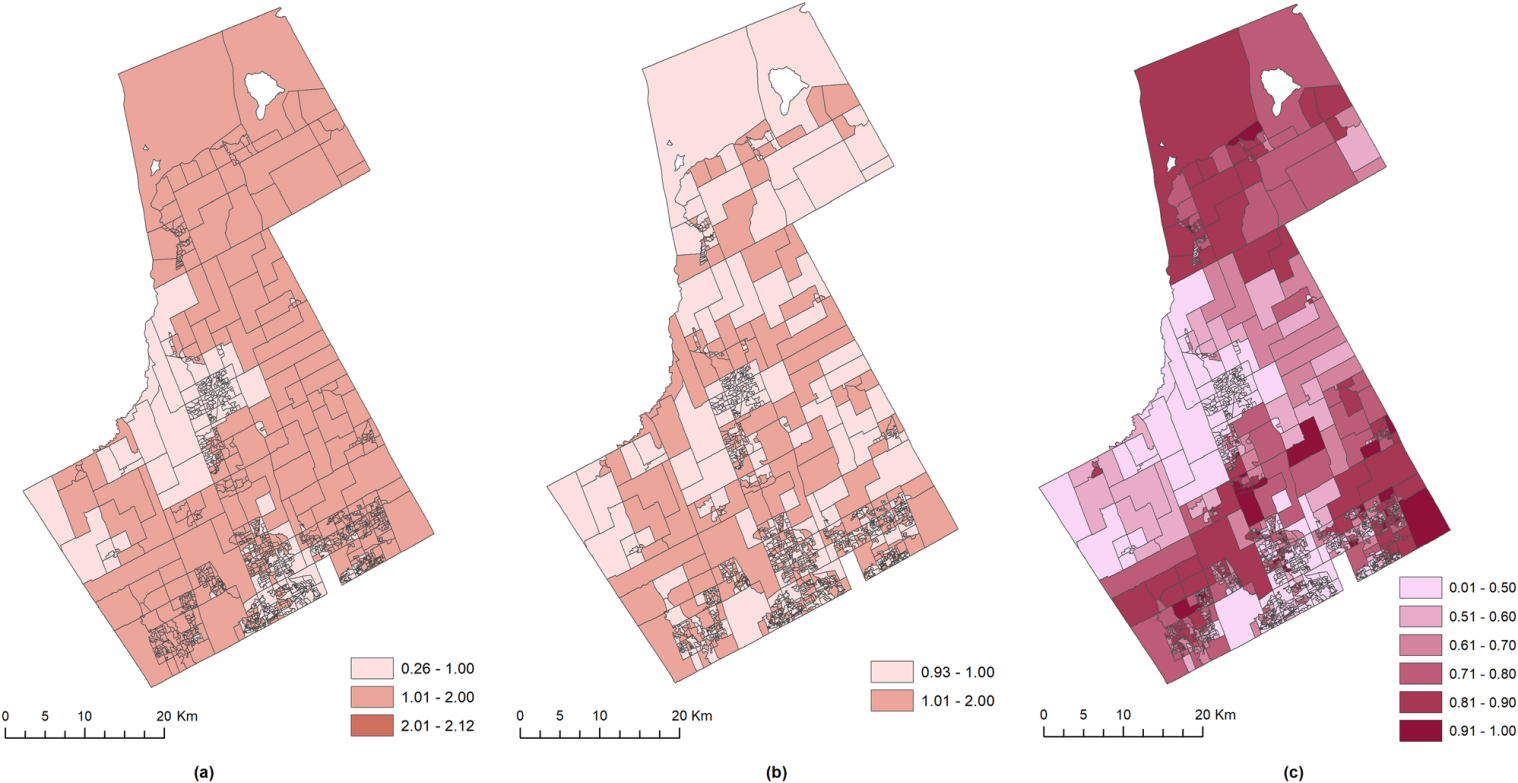
The unexplained risks that are (**a**) spatially structured $\:\left({\text{e}\text{x}\text{p}(s}_{i1}\right))$ and (**b**) non-structured $\:\left({\text{e}\text{x}\text{p}(u}_{i1}\right))$, with the hotspots of the (**c**) remaining and unexplained risk (Pr(exp($\:{s}_{i1}\:+\:{u}_{i1}$) > 1 | data)) of YO and VC

Finally, Fig. 4 illustrates the remaining (unexplained) risks of YO that are spatially structured $\:\left({\text{e}\text{x}\text{p}(s}_{i1}\right))$ and non-structured $\:\left({\text{e}\text{x}\text{p}(u}_{i1}\right))$, respectively, after controlling for the shared and socioeconomic risks. Figure 4a show that for most DAs, the unexplained risks of YO were moderately low $\:\left(1<{\text{e}\text{x}\text{p}(s}_{i1}\right)\le\:2)$ and substantially spatially structured. The non-spatial risk in Fig. 4b demonstrates a checkered pattern, suggesting that the non-spatial risk of YO did not exhibit any clustered pattern, which is in contrast to the pattern observed in Fig. 4a. Furthermore, when the probability of each DA being a hotspot of the remaining and unexplained risk (Pr(exp($\:{s}_{i1}\:+\:{u}_{i1}$) > 1 | data)) are mapped in Fig. 4c, the hotspots are mainly observed only in the northwest and southeast region of the study area.

## Discussions

This study introduces a novel application of the Bayesian shared component spatial modeling to analyze the spatial association and the spatial pattern between two outcome variables: young offenders and violent crimes. Based on the review of existing literature, the BSCS modeling technique has been predominantly used as a hotspot detection technique (Ancelet, Abellan, Del Rio Vilas, Birch, & Richardson, 2012; R. P. Haining & Li, 2020; Ibáñez-Beroiz et al., 2011; Knorr-Held & Best, 2001; Law & Perlman, 2018; Law et al., 2020; MacNab, 2010; Paradinas et al., 2017; Quick et al., 2019), and this is the first study that has extended the statistical principles of shared component modeling and applied the modeling technique for detecting the association between multiple outcomes of crime.

We aimed to jointly model offenders and offenses with linking parameters (the shared component and spatial adjacency or neighborhood effects), as such that the shared component model mimics an ecological regression on the unobserved shared component (Knorr-Held & Best, 2001). Offenders and offenses are signs of crime that, to some measure, can be exacerbated by adverse socioeconomic and built environmental factors, often displaying spatial distribution. These common spatial factors can be captured by the unobserved shared component term. The modeling technique allows offenders and offenses to be considered separate entities to identify correlations between their spatial distributions, estimate relative risk, and model the risk in terms of correlation between offenders and offenses. More specifically, the BSCS modeling approach analyzes the spatial association between YO and VC as a shared component with common data-generating processes and maps the crime-general (YO-VC shared) and the crime-specific (YO or VC specific) hotspots as areas with a high risk from YO and VC. The results suggest that the BSCS models outperformed the non-BSCS models in terms of DIC values. In particular, the BSCS model, where the potential confounders have been adjusted, can produce the best model for detecting the spatial association between YO and VC.

The findings from the BSCS model provide evidence of a significant spatial association between YO and VC. The shared component ($\:{\theta\:}_{i}$) estimated the common risks for YO and VC, and the scaling parameter ($\:\delta\:$) measured the difference between the magnitude of their area-specific relative risks. Our result of the posterior means of the scaling parameter equals 1.130 (95% CI: 0.634, 1.857), which is close to 1. This indicates that the risk gradients from YO and VC are similar and clearly indicates that YO and VC are associated. Similarly, if a study region has an overall high risk of YO but is low in VC, then the value of $\:\delta\:$ should be high, and vice versa.

The fraction of total variation in the relative risk of YO that was explained by the shared component was 0.36. This indicates that 36% of the variability of offenders is attributable to the shared component of YO and VC after controlling for ethnic heterogeneity, residential mobility, and government transfer payments. This fraction was 0.49 prior to adjusting the three significant socioeconomic risk factors that influenced the relationship between YO and VC. Therefore, this reduction in the relative contribution of the shared component to the total risk of YO indicates that future research is warranted to determine if other risk factors could be considered than the ones already used in this study. However, a major reduction of this fraction is unlikely since Model 2 indicates a significant association between YO and VC, implying that there were common hotspots or shared components for YO and VC. Moreover, the fractional risk values of the shared component provide further evidence that the YO-VC is highly spatially associated, as nearly half of the risk of YO could be explained by the risk that it shares with VC.

We observed that when the covariates were included in the BSCS model, the values for the shared risk of YO and VC were reduced for high-risk areas previously identified from the model without covariates. The shared risk maps with covariates show that in BSCS models, covariates or potential confounders could be highly beneficial to stabilize risk values, especially those at the extreme ends. This could be explained by multivariate shared component modeling principles, which exploit the correlation structures between the multiple outcomes of interests and the potential covariates (Hogan & Tchernis, 2004; Knorr-Held & Best, 2001). Therefore, including covariates that help explain the higher-order correlation between the outcomes and covariates reduces the model or process uncertainties, mainly stemming from simplifying real-life processes using statistical models (Law et al., 2014; Wikle et al., 1998). This, in turn, substantially improves the risk estimation process as the relative risk is modeled as a function of the covariates or risk factors and their unknown coefficients (such as $\:{\beta\:}_{k}$) (Law et al., 2006; Law & Quick, 2013).

In this regard, the BSCS modeling approach for detecting the spatial association between multiple outcomes, such as YO and VC, offers two specific advantages. First, the BSCS produces the shared and type-specific components of the YO and VC. Consequently, it filters out the remaining and unexplained risks of YO that are spatially structured and unstructured after controlling for the shared hotspots and socioeconomic risk factors. Therefore, any covariates that could not be measured or incorporated into the model are also adjusted during the BSCS modeling. This potentially attenuates the errors in risk estimations due to missing covariates. Second, contrary to conventional techniques such as spatial lag, spatial error, or geographically weighted regressions, BSCS modeling allows mapping the hotspots due to the unexplained risks of YO. These areas could be specifically targeted to identify the unknown risk factors of YO. For example, Fig. 4c is critically important because it explains how much risk was not explained by the YO, VC, and the three covariates. The hotspot mapping showed that closer observations are warranted for these areas as more important driving forces that were influencing the spatial distribution of YO and VC could be in action.

In contrast to past studies that have mainly focused on the relationship between victims of violent crimes and offenders (Kaukinen, 2002, 2004; Lantz, 2021), this study focused on youths as offenders. The results of both non-BSCS and BSCS modeling indicate that there is a strong association between YO and VC in an area. The criminological theories suggest that the influence of places (environmental) and surrounding people may play a major role in explaining the observed relationship (Wikström et al., 2018). The *situational action theory* indicates that the YO offenders interact with their surrounding places that govern their rule-breaking behaviors and action mechanisms (Wikström, 2006; Wikström et al., 2018). Hence, the presence of VC in the surrounding environment of an adolescent individual may act as a driving force for criminogenic inducements. The situational action theory further shows that all YO are by nature rule-abiding creatures, and the presence of VC in their surroundings impairs their personal morals, self-control, and the capacity to resist external pressures to act against their personal law-respecting beliefs (Wikström et al., 2018). Recent studies applied *rational choice* and *broken windows theories* to demonstrate that a consistent presence of VC changes social norms, causing society to become more tolerant of petty crimes and, thus, facilitating the deindividuation of crime (Lantz, 2021; Miller et al., 2016). The process thus leads to the emergence of larger groups of youths who support one another in criminal activities, enabling YO to engage in more organized and severe forms of crimes such as burglary, robbery, murder, or physical assaults.

The main strength of this study arises from the fact that the proposed BSCS modeling approach can address the immense variability in the social constructs of crime while modeling the relationship between YO and VC. In particular, several specific advantages of applying the BSCS modeling technique to detect the spatial association between multiple outcomes can be discussed. First, applying the BSCS technique transforms the conventional association analysis from a process of inputting variables as dependent and independent variables to outcome variables only. For example, using the same set of covariates, the BSCS model in Model 4 was able to jointly model the YO and VC as two outcome variables instead of one dependent (YO) and one independent variable (VC) in Model 2. Second, applying the BSCS approach transforms the existing association analysis from the results of regression coefficients to probability maps that display shared and specific geographic patterns of the outcomes. For example, it is not possible to map the relationship between YO and VC using the non-BSCS models in Models 1 and 2. However, this could be easily achieved using the BSCS models such as Model 4. As evident from Fig. 3b, the BSCS model could easily map the spatial distribution of the areas with high probabilities of YO-VC shared risks. Third, the BSCS modeling technique reduces the necessity of incorporating spatial risk factors or covariates when analyzing the relationship between multiple outcomes. Although the inclusion of covariates was found to improve the risk estimation (Fig. 2a vs. Figure 3a) and reduce the fraction of total variation in relative risk of YO that was explained by the shared component (Table 1), the association analysis could be restricted due to the availability of the relevant covariates. For example, socioeconomic data for covariates could be available at the census tract level, whereas the observed YO and VC data could be available at the neighborhood level. In such a circumstance, the BSCS modeling technique could be used to treat the YO and VC as spatially correlated outcomes with the unmeasured or missing covariates as a set of spatially structured and non-structured random effect terms. Additionally, as illustrated in Fig. 4, the unexplained risks of YO and VC due to these missing covariates could be mapped to understand the relative impact of missing covariates on risk assessments and hotspot detection. Fourth, as the BSCS modeling approach is based on the non-parametric Bayesian joint modeling framework, the association analysis does not require the outcomes to have a linear relationship. This is an advancement of current crime analysis techniques, particularly for those heavily relying on the linear relationship between YO and VC and the counts to follow a normal distribution. The linear and parametric assumptions can be easily violated for real-life processes such as YO and VC, rendering the parameter estimates unreliable.

Despite the strengths discussed, several limitations exist that should be considered while interpreting the results. First, we combined different types of VC and used aggregated counts to assess their association with YO. The association analysis can be further improved if the relationship between a specific VC and its offenders can be analyzed. For instance, if data are accessible, the analysis could benefit from separating domestic violence and violence in schools from other violent crimes. Second, male and female offenders could be differentially susceptible to VC; thus, the association with VC could be different for young male and female offenders. Hence, the modeling could be further refined if the YO data could be separated based on the sex of the offenders. However, this was not possible in our study due to the small count of female offenders in each DA, which raised concerns about a privacy breach. We found less than six female offenders in 116 of the 132 rural DAs, and, therefore, employed the total count of male and female offenders in each DA. Finally, we used the Bayesian models to analyze the YO and VC relationships from a spatial standpoint and assumed that the temporal influence of the risk factors on these outcomes has remained constant. Although temporal dynamics of YO, VC, and risk factors can play an important role in determining crime risk in an area, we have considered crime datasets that span over two years (January 1, 2006, to December 31, 2007), during which the socioeconomic and cultural risk factors can be considered to be reasonably unchanged or constant.

## Conclusions

Traditionally used spatial modeling techniques have limitations when analyzing the spatial association between complex outcomes such as young offenders (YO) and violent crimes (VC). These highly correlated outcomes influence each other in real life and are simultaneously affected by various socioeconomic confounders or risk factors. Therefore, conventional spatial regression techniques may induce model uncertainties while assuming a unidirectional relationship between the dependent and independent variables. These techniques model the spatial association between variables as a process where only the independent variables affect the distribution of the dependent variable. Furthermore, the commonly used spatial techniques do not allow the mapping of shared and crime-specific hotspots, which limits their applications in crime management practices.

Therefore, this study introduces the Bayesian shared component spatial (BSCS) modeling as a new method to analyze the geographic association and the geographic pattern between offenders (YO) and offenses (VC). Additionally, the proposed method was used to identify the crime-general (offender and offense shared), offender-specific, and offense-specific hotspots to disentangle the complex relationship between YO and VC. The spatial association between YO and VC was studied using both non-BSCS and BSCS modeling techniques, with the index of ethnic heterogeneity, residential mobility (1-year moving rate), and percentage of residents receiving government transfer payments as three socioeconomic covariates based on the preceding study by Law and Quick (2013). The results suggest that the BSCS models outperform the non-BSCS models in terms of model fit according to the deviance information criterion (DIC) values. The shared component and the scaling parameter of the BSCS models indicate a strong spatial association between the YO and VC. The fraction of total variation in the relative risk of YO that was explained by the shared component suggests that nearly half of the risk from YO in the study area was attributable to the risk shared between YO and VC. The shared risk estimation was enhanced through the inclusion of the three covariates in the BSCS models, and the remaining unexplained risks due to any unmeasured covariates were adjusted using spatial and non-spatial random effect terms. The relative risk and hotspot mapping suggested that the BSCS model with putative risk factors could stabilize risk values in areas with a high shared risk of YO and VC.

The application of the proposed BSCS modeling technique can allow the detection of spatial association between multiple outcomes interconnected through complex, non-linear, and multidirectional pathways. The proposed method, demonstrated using two outcome variables, can be extended to include more than two variables. The BSCS modeling technique allows the detection of both the spatial association and hotspots of multiple outcomes, which can be highly beneficial for targeted interventions and planning, such as managing the risks of YO and VC.

## Electronic supplementary material

Below is the link to the electronic supplementary material.

Supplementary Material 1

## Data Availability

The raw dataset analyzed in this study was provided by the York Regional Police Department, Ontario, Canada. Therefore, data-sharing restrictions apply to the analyzed dataset. The data are protected and not available due to data privacy laws.
