# Supplementary material for: A bayesian shared component spatial modeling approach for identifying the geographic pattern of local associations: a case study of young offenders and violent crimes in Greater Toronto Area
Source: Crime Sci. 2024 Oct 30;13(1):37. doi: 10.1186/s40163-024-00235-5 (PMC11525323; doi:10.1186/s40163-024-00235-5)
Supplement: Supplementary file 1 — Supplementary Material 1 [file 40163_2024_235_MOESM1_ESM.docx]

### **Models Implemented**

**Model 1:** A Bayesian Poisson log-linear model with young offenders (YO) as the outcome variable and the three covariates (the index of ethnic heterogeneity, residential mobility, and percentage of residents receiving government transfer payments).

**Model 2:** A Bayesian Poisson log-linear model with YO as the outcome variable and the above three covariates (Model 1) and violent crimes (VC) as the fourth covariate.

**Model 3:** A Bayesian shared component model with YO and VC as outcome variables and no covariates.

**Model 4:** A Bayesian shared component model with YO and VC as outcome variables and the above three covariates in Model 1.

### **Model Construction**

All the models were developed using the Bayesian framework. For this purpose, the observed counts of the YO, $O_{i}$, in each DA, *i* (where, *i* = 1, 2,…1128), were modeled using the Poisson distribution, $O_{i}\boldsymbol{\sim}Poisson(\lambda_{i})$. The parameter $\lambda_{i}$ of the distribution represented the expected value of $O_{i}$. Hence, the expected value could be modeled as a product of the area-specific expected count $E_{i}$ and the unknown relative risk, $r_{i}$, of YO in the area, which could be expressed as a function of the risk from the covariates to give the equation (Eq. 1) for Model 1. The random effect terms, $s_{i} \mathrm{and} u_{i}$, captured the spatially structured and unstructured risks, respectively, due to the unmeasured or latent covariates. Further details about modeling principles applied to Model 1 can be found in Law and Quick, 2013.

|  | ${\log[\lambda}_{i}]= {\log[E}_{i}] + \beta_{0}+ \beta_{1}X_{1i}+ \beta_{2}X_{2i}+ \beta_{3}X_{3i}+ s_{i} + u_{i}$ | (1) |
| --- | --- | --- |

Here, the $X_{1i}$, $X_{2i}$ and $X_{3i}$ represent the index of ethnic heterogeneity, residential mobility, and percentage of residents receiving government transfer payments, respectively. The $\beta_{k}$, where *k* = 1, 2, and 3, represent the regression coefficients.

Additionally, since our modeling aimed to understand whether DAs with more VC also have more YO, the VC was added as the fourth regressor (the target variable) with Eq. 1 to give the equation for Model 2 (Eq. 2).

|  | ${\log[\lambda}_{i}]= {\log[E}_{i}] + \beta_{0}+ \beta_{1}X_{1i}+ \beta_{2}X_{2i}+ \beta_{3}X_{3i}+ \beta_{4}X_{4i}+ s_{i} + u_{i}$ | (2) |
| --- | --- | --- |

Here, the $X_{4i}$represents the rate of VC in each DA and $\beta_{4}$ directly represents the magnitude and the direction (positive or negative) of the association between YO and VC.

The Bayesian shared component spatial models, Models 3 and 4, were applied to analyze the joint spatial distribution of the YO and VC (Held et al., 2005; Knorr‐Held & Best, 2001). The BSCS modeling technique is an extension of the Besag, York, and Mollie (BYM) model (Besag et al., 1991) and can jointly analyze multiple variables to give three different components of the area-specific risks of crime (Law et al., 2020):

1. **The shared component:** Explains the area-specific risk due to both YO and VC or the common risk shared between YO and VC.
2. **The type-specific component of young offenders**: Gives the area-specific risk owing to young offenders only.
3. **The type-specific component of violent crimes:** Represents the risk owing to violent crimes only.

In this study, the shared component part of the BSCS model is of particular interest for analyzing the association between YO and VC. The statistical and theoretical foundations of applying the BSCS technique for an association analysis originated from the statistical and theoretical foundations laid by Held et al. (2005) and MacNab (2010). These two studies suggested that the shared component model assumes common data-generating processes, implying that the outcomes (or variables) are correlated and could be associated due to some common data-generating (risk) factors (Held et al., 2005; MacNab, 2010).

The shared component models were also constructed using the Bayesian framework. Hence, the observed counts, $O_{ik}$ , where *k*= 1 and 2 for the YO and VC, respectively, were modeled using the Poisson distribution as:

|  | ${O_{ik}= \lambda}_{ik}= E_{ik}r_{ik}$ | (3) |
| --- | --- | --- |

We assumed that the common spatial risk shared by YO and VC in an area *i* could be given by $\theta_{i}$. In contrast, the non-shared or the type-specific risks for YO and VC could be modeled using one spatially structured random effect term, $s_{ik} ,$and one non-spatial random effect term, $u_{ik}$. Hence, using the shared and type-specific components, two separate equations could be written for the relative risk, $r_{ik}$, owing to YO and VC:

|  | $r_{i1}$= exp $(\alpha_{1}+ \delta\theta_{i}+s_{i1}+ u_{i1})$ | (4) |
| --- | --- | --- |
|  | $r_{i2}$= exp ${(\alpha}_{2}+\frac{1}{\delta}\theta_{i}+s_{i2}+ u_{i2})$ | (5) |

Here, $\alpha_{1}$ and $\alpha_{2}$ are the specific intercepts for YO and VC, respectively, and give the baseline or average risks from YO or VC in an area. Finally, Eq. 3, 4, and 5 were integrated and log-transformed to develop the equations for BSCS models (Models 3 and 4). Eq. 6 and 7 were used for constructing Model 3.

|  | ${log ( \lambda_{i1}})=\log{(E}_{i1})$ + $\alpha_{1}+ \delta\theta_{i}+s_{i1}+ u_{i1}$ | (6) |
| --- | --- | --- |
|  | ${log ( \lambda_{i2}})=\log{(E}_{i2})$ + $\alpha_{2}+\frac{1}{\delta}\theta_{i}+s_{i2}+ u_{i2}$ | (7) |

The unique contribution from YO and VC in the shared risk and the risk gradient of the shared component was modeled as a scaling parameter $\delta$ (where $\delta$ > 0). Moreover, to enhance the model identifiability, the VC was assigned with an inverse of the scaling parameter used for YO in Eq. 8 (Knorr‐Held & Best, 2001; Lawson, 2009). Thus, a value of $\delta$ near to one would indicate that the YO and VC have similar contributions to the shared pattern. Contrastingly a large positive value of $\delta$ would indicate that the YO contributed more to the shared risk than the VC and has a stronger spatial association with the shared component compared to VC.

Eq. 6 and 7 were further modified to include covariates or putative risk factors that may influence the distribution of YO and VC in the study area. Consequently, the $r_{ik}$ can be considered a function of the shared and type-specific risks and the risks owing to the influence of covariates.

|  | ${log ( \lambda_{i1}})=\log{(E}_{i1})$ + $\alpha_{1}+ \delta\theta_{i}+ \beta_{0}+ \beta_{1}X_{1i}+ \beta_{2}X_{2i}+ \beta_{3}X_{3i}+s_{i1}+ u_{i1}$ | (8) |
| --- | --- | --- |
|  | ${log ( \lambda_{i2}})=\log{(E}_{i2})$ + $\alpha_{2}+\frac{1}{\delta}\theta_{i}+ \beta_{0}+ \beta_{1}X_{1i}+ \beta_{2}X_{2i}+ \beta_{3}X_{3i}+s_{i2}+ u_{i2}$ | (9) |

Eq. 8 and 9 were used to construct Model 4, which is the main model of interest in this study. Additionally, based on the joint modeling concepts (Baker et al., 2017; Haining & Li, 2020; Martins et al., 2016), Model 4 was considered to mimic an ecological regression, where YO functions as an outcome, VC represents a latent covariate (modeled as a part of the shared component with YO), and the three covariates operate as the independent variables. Under such circumstances, the $\theta_{i}$ with the scaling parameter $\delta$ would directly correspond to the magnitude of the association between YO and VC. A risk gradient ($\delta$) closer to 1 would be indicative of YO and VC equally contributing to the $\theta_{i}$ and therefore, having common data-generating processes that would cause the two outcomes to be strongly associated (Held et al., 2005; Knorr‐Held & Best, 2001; Law et al., 2020; MacNab, 2010). Alternatively, a high value of the risk gradient would imply that the YO has contributed more to the shared component than VC and vice versa, suggesting less commonality between the two outcomes.

It is important to note that by virtue of the shared component concept, in our association modeling, the shared component will only indicate a positive association between the studied variables (here, YO and VC). From a risk management perspective, outcomes that are positively associated with the risk are of primary concern, as controlling these outcomes assists in risk mitigation. However, the no (positive) association modeled by the BSCS approach indicates two types of possible scenarios: no/zero association and negative association. For example, areas with only a high risk of YO indicate that the area has a high occurrence of YO and a low occurrence of VC. This implies that the YO has no relationship with VC in the area (zero association) or YO has an inverse (negative association) relationship. Therefore, although the shared component in our model provides information about the positive association between two outcomes of interest, the non-shared or outcome-specific components offer information about non-positive or zero and negative associations.

### **Model Implementation and Assessment Criteria**

Defining the prior distributions of unknown parameters is an integral part of developing Bayesian models (Haining & Li, 2020). Therefore, various prior distributions were used based on the nature of the parameter to be estimated. As the non-BSCS models were adopted from Law and Quick (2013), the details on prior distribution for non-BSCS models could be found in Law and Quick (2013) and Law et al. (2006).

For the BSCS models, we applied non-informative prior distributions as required. The coefficients $\beta_{1}, \beta_{2}, \beta_{3}$ and $\beta_{4}$ were specified using the normal distribution with an expected mean of 0 and a precision of 0.001. However, an improper uniform prior, dflat() prior, was assigned to the intercept $\beta_{0}$ . Contrastingly, the intercepts, $\alpha_{1}$ and $\alpha_{2}$, were set to have normal priors and the intrinsic normal conditional autoregressive (ICAR) prior distribution was used for the spatially structured random effect terms, $\theta_{i}, s_{i1} \mathrm{and} s_{i2}$. In the ICAR, the neighborhood weight information was employed for defining the conditional prior distributions of the three spatial random effect terms (Besag et al., 1991; Law & Haining, 2004). For example, the conditional distribution of the shared component $\theta_{i}$ was defined as:

|  | $\theta_{i}\vert\theta_{j}= \theta_{j}, j\neq i, j is a neighbor of i \sim N (\bar{\theta}_{i}, \frac{\omega_{\theta}^{2}}{m_{i}})$ | (11) |
| --- | --- | --- |

Where, $\theta_{i}|\theta_{j}$ is given by the conditional distribution with mean $\bar{\theta}_{i}= \sum_{j\neq i} \omega_{i,j}\theta_{j}/ m_{i}$ and the variance $\frac{\omega_{\theta}^{2}}{m_{i}} .\left\{ \omega_{i,j} :i,j=1,2, \ldots, n \right\}$, being defined by a 0-1 contiguity matrix (**W**). Here, $\omega_{i,j}=1$ for cases where *i* and *j* are neighbors and for non-neighboring cases $\omega_{i,j}=0$ and $\omega_{i,i}=0$. The $m_{i}$ represents the sum of neighbors in an area and is given by $\sum_{j} \omega_{i,j}$.

Furthermore, we used Gamma (0.1, 0.1) as the prior for the precision parameters of $\theta_{i},s_{i1}, \mathrm{and} s_{i2}$. In contrast, a normal prior distribution with mean zero and a hyperparameter precision of Gamma (0.01, 0.01) was used for the unstructured random effect terms, $u_{i1} \mathrm{and} u_{i2}$. The hyperparameter selection for both the spatial and non-spatial random effect terms was based on the recommendation of Ancelet et al. (2012) (Ancelet et al., 2012). Similarly, based on the study of Knorr-Held and Best (2001), we assumed that both the scaling parameters ($\delta$ and $\frac{1}{\delta}$ ) in Eq. 9 and 10 are non-zero, and positive and the ratio of risk gradients ranges between 0.2 and 5 (Knorr‐Held & Best, 2001). Consequently, we applied a normal prior with a mean of 0 and a precision of 5.9 (variance = 0.17) for the $\log(\delta)$ parameter in the model. It is worth mentioning that although the values of estimates from Bayesian models may be slightly sensitive to the choice of priors (Held et al., 2005), the effect could be considered negligible for the main results of the shared component spatial models (Ancelet et al., 2012; Ibáñez-Beroiz et al., 2011).

All the Bayesian models in this study were fitted using the WinBUGS software (Lunn et al., 2009). The model convergence was achieved using two Markov Chain Monte Carlo (MCMC) chains initiated at dispersed starting values. The Monte Carlo (MC) standard error of the posterior mean for each parameter was used for the accuracy assessments and to determine whether sufficient samples had been taken after the model convergence (Law et al., 2006). The number of samples was considered adequate when the MC error for each parameter was <5% of the sample posterior standard deviation.

The deviance information criterion (DIC) for each model was used to evaluate and select the best model. As the DIC considers both the model fit (*D*) and the model complexity ($p_{D}$), an increase in the number of effective parameters would increase the model complexity and prevent the development of a parsimonious model. Ideally, the best model should be parsimonious and have a DIC value that is at least five units smaller than the DIC values of other models (Law et al., 2015).

### **References**

Ancelet, S., Abellan, J. J., Del Rio Vilas, V. J., Birch, C., & Richardson, S. (2012). Bayesian shared spatial‐component models to combine and borrow strength across sparse disease surveillance sources. *Biometrical Journal, 54*(3), 385-404.

Baker, J., White, N., Mengersen, K., Rolfe, M., & Morgan, G. G. (2017). Joint modelling of potentially avoidable hospitalisation for five diseases accounting for spatiotemporal effects: A case study in New South Wales, Australia. *PloS One, 12*(8), e0183653.

Besag, J., York, J., & Mollié, A. (1991). Bayesian image restoration, with two applications in spatial statistics. *Annals of the institute of statistical mathematics, 43*(1), 1-20.

Haining, R. P., & Li, G. (2020). *Modelling Spatial and Spatial-Temporal Data: A Bayesian Approach*. CRC Press.

Held, L., Natário, I., Fenton, S. E., Rue, H., & Becker, N. (2005). Towards joint disease mapping. *Statistical Methods in Medical Research, 14*(1), 61-82.

Ibáñez-Beroiz, B., Librero-López, J., Peiró-Moreno, S., & Bernal-Delgado, E. (2011). Shared component modelling as an alternative to assess geographical variations in medical practice: gender inequalities in hospital admissions for chronic diseases. *BMC Medical Research Methodology, 11*(1), 1-10.

Knorr‐Held, L., & Best, N. G. (2001). A shared component model for detecting joint and selective clustering of two diseases. *Journal of the Royal Statistical Society: Series A (Statistics in Society), 164*(1), 73-85.

Law, J., & Haining, R. (2004). A Bayesian approach to modeling binary data: The case of high‐intensity crime areas. *Geographical Analysis, 36*(3), 197-216.

Law, J., Haining, R., Maheswaran, R., & Pearson, T. (2006). Analyzing the relationship between smoking and coronary heart disease at the small area level: a Bayesian approach to spatial modeling. *Geographical Analysis, 38*(2), 140-159.

Law, J., Quick, M., & Chan, P. W. (2015). Analyzing hotspots of crime using a Bayesian spatiotemporal modeling approach: a case study of violent crime in the Greater Toronto Area. *Geographical Analysis, 47*(1), 1-19.

Law, J., Quick, M., & Jadavji, A. (2020). A Bayesian spatial shared component model for identifying crime-general and crime-specific hotspots. *Annals of GIS*, 1-15.

Lawson, A. (2009). Multivariate Disease Analysis. *Bayesian Disease Mapping: Hierarchical Modeling in Spatial Epidemiology, 1st ed.; Keiding, N., Morgan, BJT, Wikle, CK, van der Heijden, P., Eds*, 206.

Lunn, D., Spiegelhalter, D., Thomas, A., & Best, N. (2009). The BUGS project: Evolution, critique and future directions. *Statistics in Medicine, 28*(25), 3049-3067.

MacNab, Y. C. (2010). On Bayesian shared component disease mapping and ecological regression with errors in covariates. *Statistics in Medicine, 29*(11), 1239-1249.

Martins, R., Silva, G. L., & Andreozzi, V. (2016). Bayesian joint modeling of longitudinal and spatial survival AIDS data. *Statistics in Medicine, 35*(19), 3368-3384.
